# Antibacterial effect of vitamin C against uropathogenic *E. coli *in vitro and in vivo

**DOI:** 10.1186/s12866-023-02856-3

**Published:** 2023-04-20

**Authors:** Noha Anwar Hassuna, E. M. Rabie, W. K. M. Mahd, Marwa M. M. Refaie, Rehab Kamal Mohamed Yousef, Wedad M. Abdelraheem

**Affiliations:** 1 Medical Microbiology and immunology department- Faculty of Medicine, Minia University, Minia, Egypt; 2 Department of Pharmacology, Faculty of Medicine, Minia University, Minia, Egypt; 3 Department of Pathology, Faculty of Medicine, Minia University, Minia, Egypt

**Keywords:** *E. coli*, UTI, Vitamin C, Antibacterial, Anti-biofilm, Nitrofurantoin

## Abstract

**Background:**

Resistance to antibiotics has increased steadily over time, thus there is a pressing need for safer alternatives to antibiotics. Current study aims to evaluate the influence of vitamin C as an antibacterial and anti-biofilm agent against uropathogenic *E. coli* (UPEC) strains. The expression of beta-lactamases and biofilm encoding genes among *E. coli* isolates before and after treating the isolates with sub MIC of vitamin C was analyzed by Real-time PCR. The in vivo assessment of the antibacterial and anti-biofilm effects of vitamin C against uropathogenic *E. coli* strains was done using a urinary tract infection (UTI) rat model.

**Results:**

The effective concentration of vitamin C that could inhibit the growth of most study isolates (70%) was 1.25 mg/ml. Vitamin C showed a synergistic effect with most of the studied antibiotics; no antagonistic effect was detected at all. Vitamin C showed an excellent anti-biofilm effect against studied isolates, where 43 biofilm-producing isolates were converted to non-biofilm at a concentration of 0.312 mg/ml. The expression levels of most studied genes were down-regulated after treatment of *E. coli* isolates with vitamin C. In vivo assessment of vitamin C in treating UTIs showed that vitamin C has a rapid curative effect as the comparable antibiotic. Administration of both vitamin C and nitrofurantoin at a lower dose for treatment of UTI in rats had a better effect.

**Conclusion:**

Vitamin C as an antibacterial and anti-biofilm agent either alone or in combination with antibiotics could markedly improve UTI in experimental rats.

**Supplementary Information:**

The online version contains supplementary material available at 10.1186/s12866-023-02856-3.

## Background

Antibiotic resistance is a major concern that is becoming a global problem worldwide due to the fast evolution of genetic exchange among bacterial species, so most potent antibiotics rapidly lose their efficacy. Biofilm is a group of microorganisms that attach to a surface forming a three-dimensional structure containing a protective polymeric matrix, which renders them highly resistant to antibiotics [1]. Some *E. coli* strains have been thought to employ the biofilm-forming abilities as a virulence factor to overcome the immune system and antimicrobial challenges [2]. Biofilm could also reduce antibiotic penetration into bacterial cells, give them a slower metabolic rate, and easier exchange of resistance genes among cells [3].

There is a pressing need for safer alternatives for antibiotics, particularly those that are natural, non-toxic, and do not cause resistance. Vitamin C (Ascorbic acid), a natural antioxidant component, is one such option that has no side effects, inexpensive, and is easily accessible [4]. Vitamin C is an essential water-soluble vitamin that cannot be synthesized by the body so it must be supplemented daily [5]. Vitamin C can be found in green peppers, red peppers, strawberries, tomatoes, broccoli, brussels sprouts, turnip, Indian gooseberry, and other leafy vegetables. However, animal sources are poor in vitamin C [6]. It is easily oxidized and damaged by oxygen and alkali; high temperature can also do this. It can be synthesized by most plant and animal species from glucose and galactose through the uronic acid pathway but humans and other primates cannot do so because of the lack of enzyme gluconolactone oxidase required for its biosynthesis [7]. The aim of this study is in vitro and in vivo evaluation of vitamin C as an antibacterial and anti-biofilm agent alone and in combination with antibiotics against uropathogenic *E. coli* strains, also to analyze the effect of vitamin C on the expression of certain antibiotic resistant genes and biofilm encoding genes of *E. coli* isolates.

## Results

### Antimicrobial susceptibility testing of antibiotics

The results of the susceptibility testing of all *E. coli* strains were categorized into sensitive, intermediate, and resistant strains according to CLSI breakpoints. The results were presented in Table 1. The most resistant phenotypes of our isolates were cefazolin, cefoxitin, and levofloxacin and the most sensitive phenotypes were meropenem and ampicillin-sulbactam.

**Table 1:** Antimicrobial susceptibility pattern of *E. coli* isolates:

Antibiotic	Total *N* = 100 (100%)		
	**Resistant** **N (%)**	**Intermediate** **N (%)**	**Sensitive** **N (%)**
**Meropenem**	20 (20%)	31 (31%)	49 (49%)
**Levofloxacin**	56 (56%)	10(10%)	34 (34%)
**Ceftazidime**	55 (55%)	12 (12%)	33 (33%)
**Nitrofurantoin**	53 (53%)	9 (9%)	38 (38%)
**Ampicillin-sulbactam**	42 (42%)	14 (14%)	44 (44)
**Cefazolin**	90 (90%)	0 (0%)	10 (10%)
**Cefoxitin**	88 (88%)	4 (4%)	8 (8%)

### Antibacterial effect of vitamin C

MIC of vitamin C against all study isolates was determined by the micro-dilution method. Out of 100 *E. coli* isolates, 15 isolates were inhibited by vitamin C at a concentration of 0.625 mg/ml, 70 isolates were inhibited by vitamin C at a concentration of 1.25 mg/ml and 15 isolates were inhibited by vitamin C at a concentration 2.5 mg/ml. The most effective concentration of vitamin C against most study isolates was 1.25 mg/ml as it can inhibit most *E. coli* isolates. There was no difference between MIC of vitamin C of antibiotic-sensitive or antibiotic-resistant strains.

### The combined effect of vitamin C with antibiotics

The results of the combined effect of Vitamin C with antibiotics were summarized in Table 2. FIC calculation showed that vitamin C had a synergistic effect with most antibiotics (Levofloxacin, Meropenem, Ceftazidime and Nitrofurantoinm). Ampicillin/sulbactam, Cefazolin, and Cefoxitin showed an indifference effect when combined with vitamin C, no antagonistic effect was detected.

**Table 2:** Fractional inhibitory concentration (FIC) of antibiotics and vitamin C

Antibiotic + vitamin C (A + B)	FIC_A_	FIC_B_	FICI	Result
Meropenem + Vitamin C	0.125	0.25	0.375	Synergism
Levofloxacin + Vitamin C	0.125	0.25	0.375	Synergism
Ceftazidime + VItamin C	0.25	0.25	0.5	Synergism
Nitrofurantoin + Vitamin C	0.25	0.25	0.5	Synergism
Amp/Sulbactam + Vitamin C	0.5	0.25	0.75	Indifference
Cefazolin + Vitamin C	1	0.25	1.25	Indifference
Cefoxitin + Vitamin C	1	0.25	1.25	Indifference

*FICA* FIC of antibiotic, *FIC B* FIC of vitamin C, *FICI* FIC index, The FIC index value ≤ 0.5 indicates synergy, > 0.5–4 indifference, and > 4 antagonism

### Anti-biofilm effect of vitamin C

All *E. coli* isolates were tested for biofilm formation before and after in vitro treatment with vitamin C at different concentrations. Out of 100 uropathogenic *E. coli* isolates, 74 isolates were recorded as biofilm produced by the MTP method. Vitamin C had an anti-biofilm effect on study isolates. The biofilm inhibitory concentration (BIC) of each isolate was determined. The most effective concentration of vitamin C against most biofilm-producing isolates was 0.312 mg/ml (Supplementary Fig. S1) where 43 biofilm-producing isolates were converted to non-biofilm at this concentration, 24 isolates were converted to non-biofilm at a concentration of 0.625 mg/ml, and 7 isolates were converted to non-biofilm at a concentration of 1.25 mg/ml of vitamin C.

### Determination of MIC_50_ and MIC_90_, BIC_50_, and BIC_90_ of the isolates

MIC_50_ and MIC_90_ values as well as the range of values obtained are important parameters for reporting results of susceptibility testing when multiple isolates of a given species are tested. The MIC_50_ represents the MIC value at which 50% of the isolates in a test population are inhibited. The MIC_90_ represents the MIC value at which 90% of the strains within a test population are inhibited. MIC_90_ was a concentration of 2.5 mg/ml of vitamin C and MIC_50_ was a concentration of 1.25 mg/ml of vitamin C. On the other hand, BIC_90_was a concentration of 0.625 mg/ml of vitamin C, and BIC _50_ was a concentration of 0.312 mg/ml.

### Time kill-kinetics assay

**Fig. 1:**
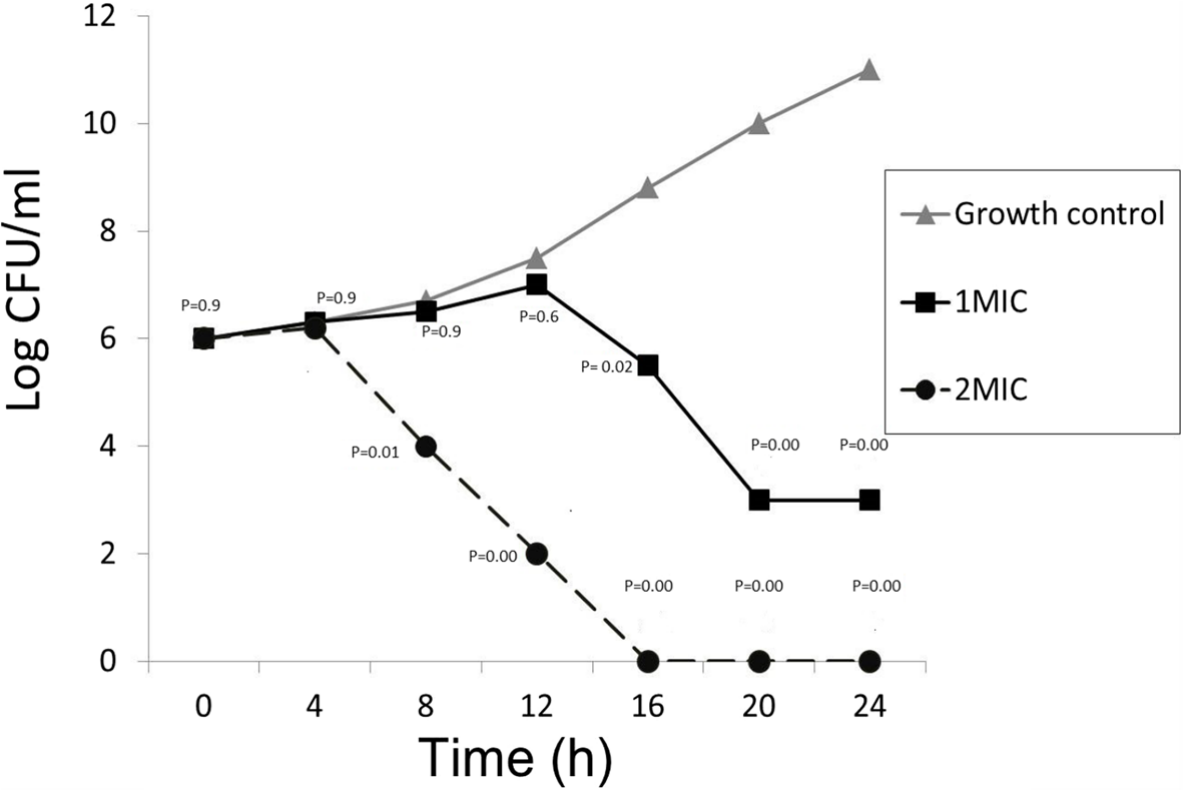
Time kill kinetics of vitamin C at 1MIC and double MIC: Mean from 3 replicates plotted for all panels; P is the *P* value, differences were considered significant when the *P* value < 0.05

The time-kill kinetics study of the 2 MIC of vitamin C against the tested *E. coli* isolates significantly reduced the number of viable bacterial cells over the first 20 and 24 h, respectively. Moreover, the double MIIC of vitamin C significantly reduced the number of viable bacterial cells over the first 12, 16, 20, and 24 C(*P* value <0.05) as shown in Fig. 1.

### Effect of vitamin C on the expression of antibiotic resistant genes and biofilm encoding genes

Real-time PCR was used for assessing the effect of vitamin C on the expression level of beta-lactamases encoding genes (*bla*_*SHV*_*, bla*_*TEM,,*_* bla*_*CTX-M,*_* bla*_*VIM,,*_*bla*_*NDM,*_* and bla*_*IMP*_) and biofilm-associated genes (*las*R, *lec*A and *pel*A), respectively before and after vitamin C treatment. The median and the interquartile range (IQR) of the expression level for the studied genes before and after vitamin C treatment were presented in Table 3. The expression levels of all studied genes were decreased after treatment of *E. coli* isolates with vitamin C. Vitamin C significantly down regulated the expression level of the following genes: *bla*_*SHV*_*, bla*_*TEM,*_* bla*_*CTX-M,,*_* bla*_*NDM,*_* bla*_*IMP,*_* las*R, and *lec*A (*P* value: 0.005, 0.003, 0.012, 0.007, 0.005, 0.028, and 0.013 respectively)*.* However, the reduction in the expression level of *bla*_*VIM*_* and pel*A genes after treating *E. coli* isolates with vitamin C was statistically not significant (*P* value: 0.059 and 0.333 respectively).

**Table 3:** Gene expressions fold change before and after treatment of *E. coli* isolates with vitamin C

**Gene**	**Before vitamin C treatment** Median (IQR**)**	**After vitamin C treatment** Median (IQR)	***P*-value**
*bla*_*SHV*_	986.8 (699.1–12,284.4)	259.5 (85.6–877.7)	0.005*
*bla*_*TEM*_	127.5 (101.1–183.1)	20.7 (11.5–68.6)	0.003*
*bla*_*CTX-M*_	115.9 (82.7–191.6)	32.7 (11.2–64.1)	0.012*
*Bla*_*VIM*_	1107.4 (814.8–2269.4)	1023.3 (755–1813.1)	0.059
*bla*_*NDM*_	70.1 (48.2–125.1)	14.4 (5.7–30.2)	0.007*
*bla*_*IMP*_	100.8 (80.4–207)	9.4 (3.8–24.3)	0.005*
*LasR*	1377.6 (269.1–5762.5)	359.4 (170.4–575.7)	0.028*
*LecA*	3381.3 (1395.8–8169.8)	202.7 (87–2514.9)	0.013*
*PelA*	4480.9 (398–6655.8)	2283.8 (849.7–4755.2)	0.333

**-**Wilcoxon Signed Rank test was used for comparison of non-parametric quantitative data between the two times, *: Significant level at *P*- value < 0.05

### In vivo assessment of the antibacterial effect of vitamin C

#### Bacterial count

In our study urine samples from rat receiving vitamin C alone or in combination were highly acidic (PH = 4–5), the group receiving nitrofurantoin alone were also acidic but lower than that receiving vitamin C (PH = 6–7) and urine samples of infected not treated groups were mostly neutral (PH = 7).

**Fig. 2:**
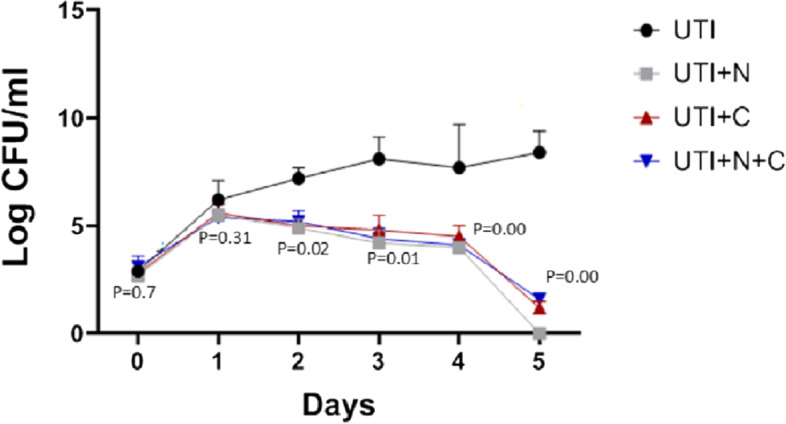
Bacterial count in the urine of different rat groups, P is the *P* value of treatment groups compared to UTI group, differences were considered significant when the *P* value < 0.05

**Fig. 3:**
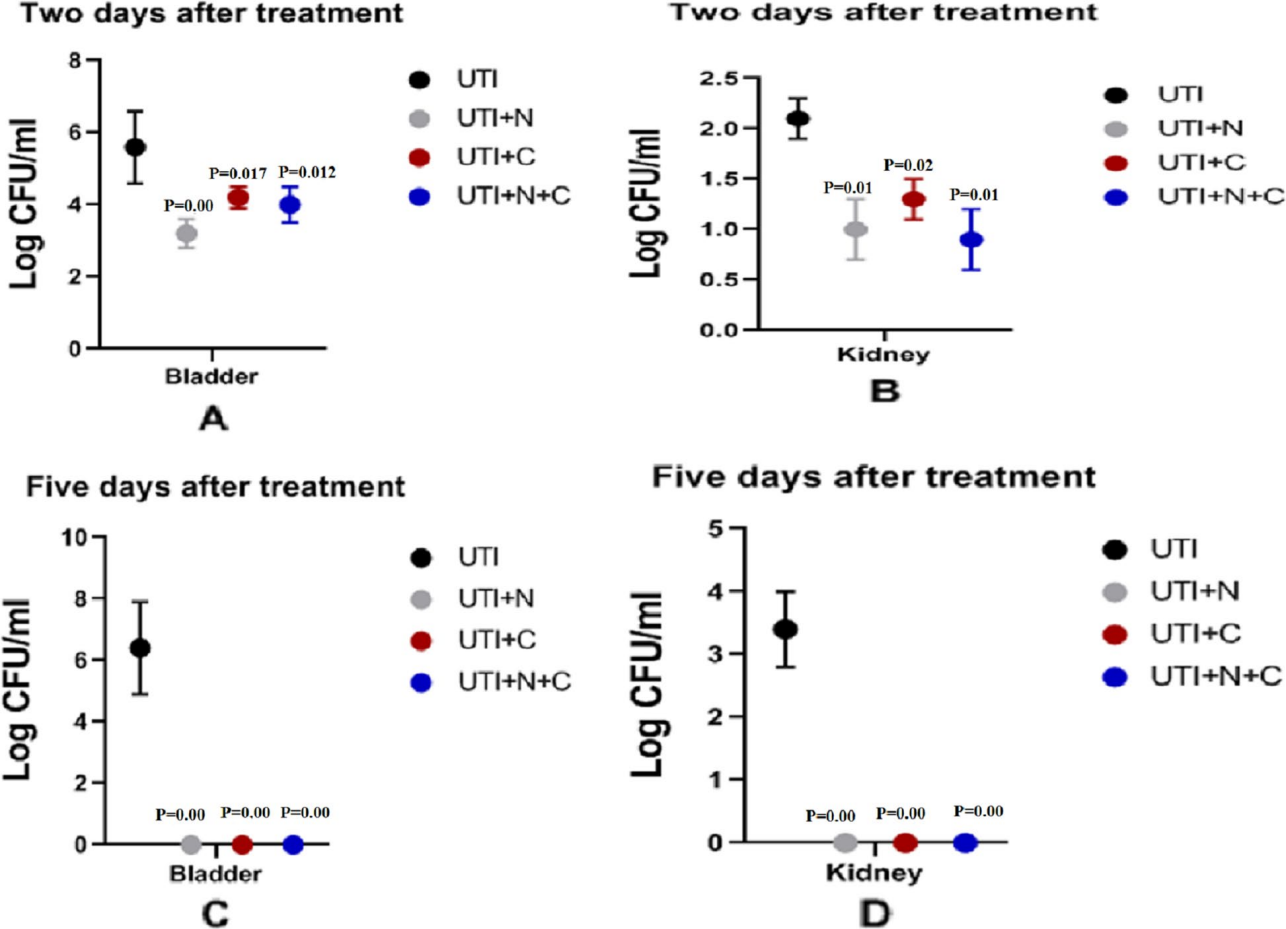
Bacterial count in the Kidney and urinary bladder of different rat groups. **A** and **B**, bacterial count in bladder and Kidney of different rat groups, 2 days after treatment; C and D Bacterial count in bladder and Kidney of different rat groups, five days after treatment. P is *P* value of treatment groups compared to UTI group, differences were considered significant when the *P* value < 0.05

The bacteria were counted in the urine at day zero and then daily after the start of treatment in all treatment groups and the positive control (UTI) group. There was a significant reduction of bacterial counts in urine in all treatment groups in comparison with the control group (*P* value <0.05) started from the first day after treatment and thereafter as shown in Fig. 2. Five days after treatment, the reduction of bacterial counts in urine was more pronounced in all treated groups. Two days after treatment and at the end of the experiment we examined the bacterial count in the urinary bladder and kidney of all groups. We found a significantly higher average CFU of bladder and kidney in rats of the UTI group than in rats of all treated groups as shown in Fig. 3. Five days after treatment, Kidney and urinary bladder returned sterile for all treatment groups.

#### Evaluation of oxidative stress parameters (MDA, GSH, TAC) and NFkB

**Fig. 4:**
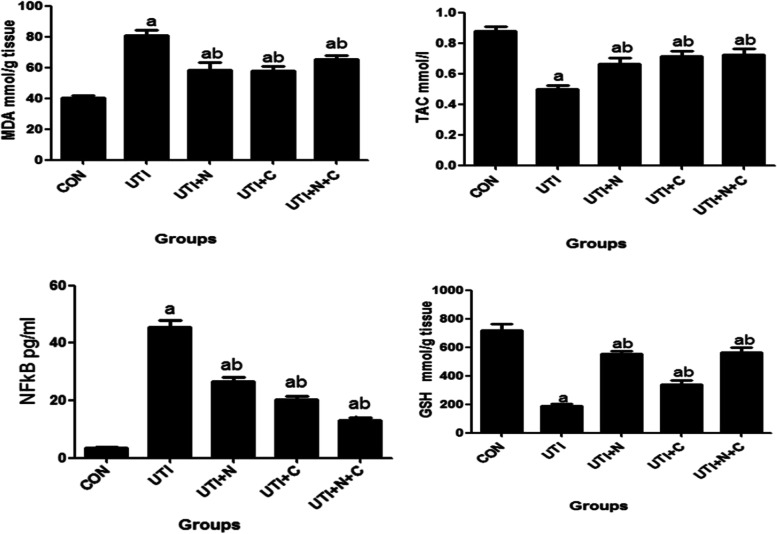
Oxidative stress parameters of different rat groups: ^s^ignificant difference of treatment groups compared to control group. ^b^significant difference of treatment groups compared to UTI group. Differences were considered significant when the *P* value < 0.05

Our data revealed that the UTI group showed significant increases in MDA, and NFkB but significant decreases in GSH, and TAC compared to the control group. However, UTI + N, UTI + C, and UTI + N + C showed significant decreases in MDA, and NFKB but increases in GSH, and TAC compared to the UTI group as shown in Fig. 4.

#### Histological examination

**Fig. 5:**
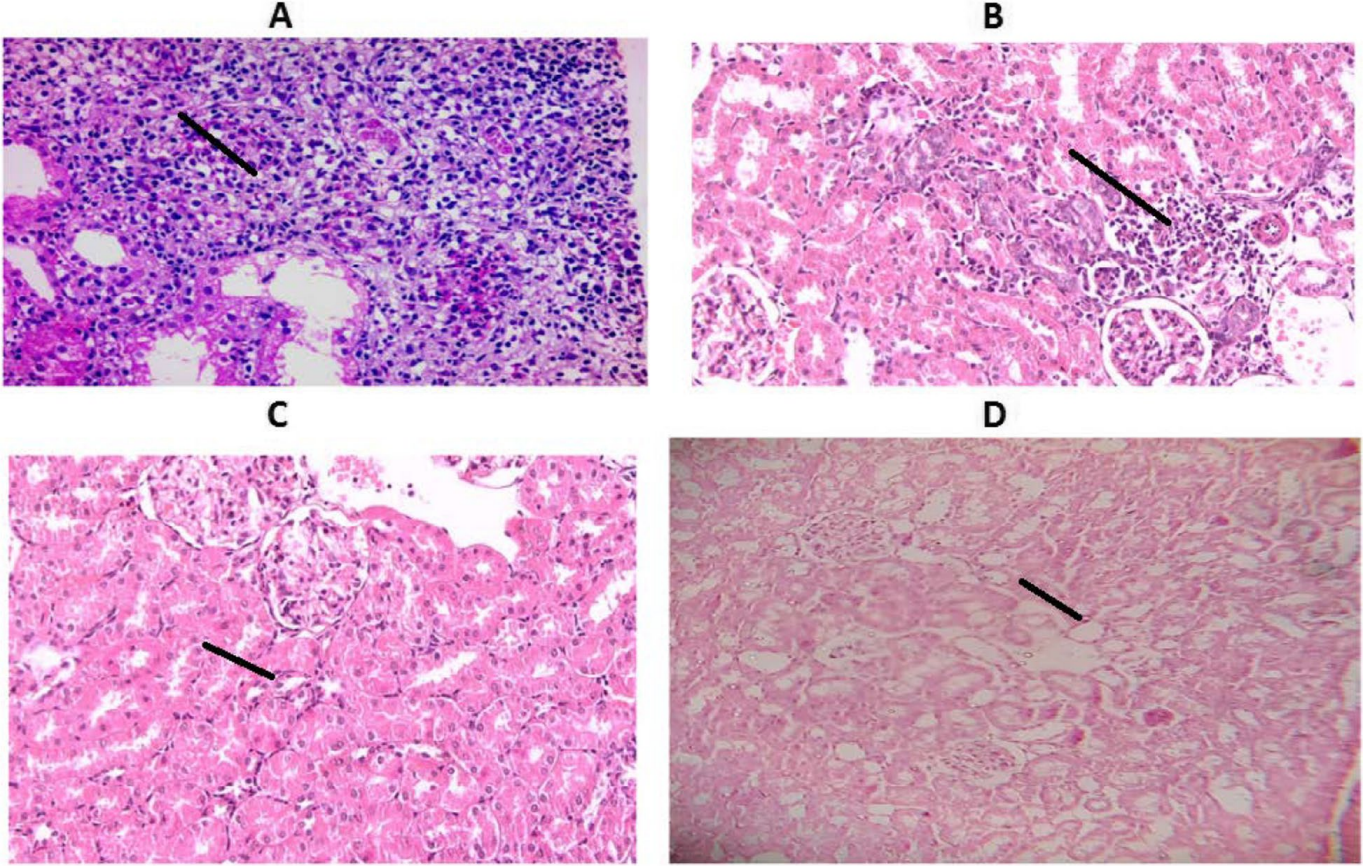
Histological picture of rat kidney tissue. Histopathology was taken on the second day after treatment or forth day after *E. coli* inoculation. **A** UTI group showed severe inflammation and marked inflammatory infiltrates. **B** UTI + C group showed mild inflammation in the form of infiltration of the interstitial tissue with inflammatory cells (black arrow). **C** UTI + N group showed cloudy swelling (a mild form of cell injury) of the tubules with a closed lumen (black arrow). **D** UTI + N + C group there is decreased cloudy swelling with a reformation of the lumen (black Arrow) and absence of inflammation

**Fig. 6:**
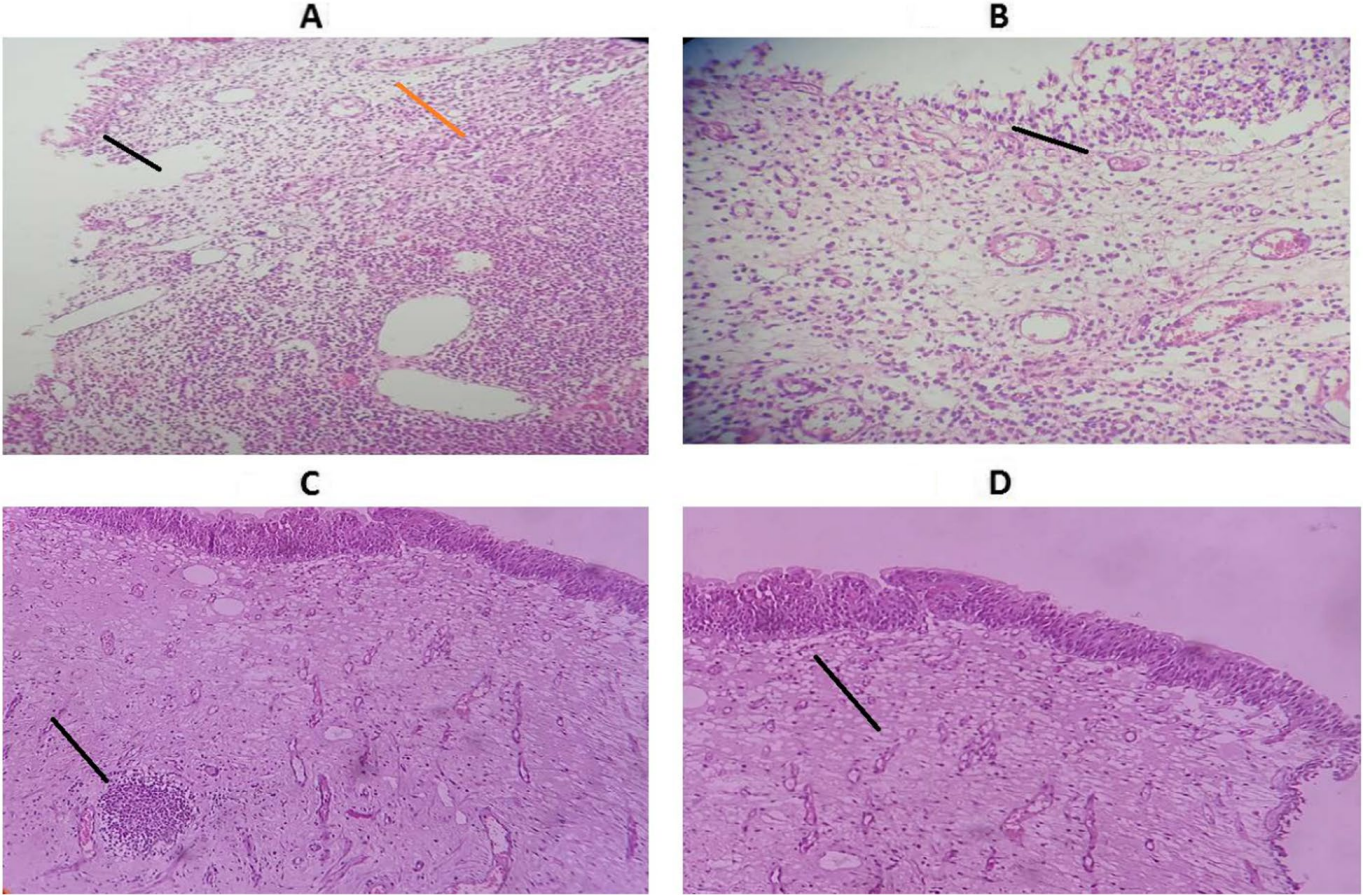
Histological picture of rat urinary bladder. Histopathology was taken on the second day after treatment or forth day after *E. coli* inoculation. **A** UTI group showed ulceration of the surface epithelium (black arrow) with intense infiltration with inflammatory cell (red arrow); **B** UTI + C group showed mild infiltration with inflammatory cells; **C** UTI + N group showed infiltration of the mucosa by collection of inflammatory cells (black arrow); **D** UTI + N + C group showed infiltration of mucosa with few scattered inflammatory cells

A comparison of the histological picture of rat kidney and urinary bladder of different groups was presented in figs. 5 and 6 respectively. Significant differences were found in kidney histopathology between the UTI group and the other treated groups. The kidneys of rats in the UTI group, killed 4 days after *E. coli* inoculation revealed severe signs of inflammation. In all treated rats (killed 2 days after treatment), either by antibiotic, vitamin c, or both, there was significant improvement of the histological picture and a significant reduction in the inflammatory infiltrates.

## Discussion

In this study, *E. coli* isolates showeda higherresistant rate to cefazolin (90%), levofloxacin (56%), Ceftazidime (55%), Nitrofurantoin(53%), and ampicillin-sulbactam (42%). These results were in compatible with other previous studies [8–11].

The higher antibiotic resistance observed in this study may be due to emergence of resistant strains and excessive unwanted use of antibiotics so we recommended continuous follow of antibiotic resistance to catch the new resistant strains, requesting un needed antibiotics should be avoided and antibiotic susceptibility before antibiotics prescription should be done.

Although carbapenems were thought to be the best treatment for MDR *E. coli* infections, resistance to carbapenems is spreading due to the acquisition of *β*-lactamase, and carbapenemase [12]. Meropenem resistance in the present study was 20% among all *E. coli* isolates. Abd El-Baky et al*.* observed that *E. coli* in Egypt showed a 13% resistance rate to meropenem [13].

New multidrug-resistant bacteria strains are constantly developing. So, alternative therapeutic modalities that can aid in the control of the antibiotic resistance pandemic must be investigated, one such possibility is vitamin C [14].

Vitamin C's antibacterial effect is influenced by bacterial strain and concentration. The antibacterial properties of vitamin C may be due to its acidic PH. The plasma level of Vitamin C is influenced by daily consumption. Higher doses (> 100 mg/day) raise the plasma vitamin C levels and then eliminated almost entirely in the urine [15]. Urine excretion of vitamin C is significantly higher in persons taking a 2000 mg dosage when compared to 100 mg of vitamin C per day [16]. So, vitamin C provided considerable protection against uropathogenic adhesion and colonization of microbes that infect the urinary tract [17] through a high vitamin C consumption that tends to raise urine acidity, which is not well tolerated by the bacteria that cause UTIs [18]. Vitamin C is a reducing agent capable of rapidly eliminating reactive oxygen species (ROS), it also works as a pro-oxidant antibacterial molecule, potentially altering the antimicrobial efficacy of some antibiotics by two proposed mechanisms: I- the transfer of vitamin C into bacterial cells, which results in the synthesis of H2O2 and, as a result, ROS; and II- The production of lactate and acetic acids from vitamin C. In addition, the interaction of vitamin C with catalytically active metal ions could contribute to oxidative damage [19].

In our study, it was found that vitamin C showed excellent antibacterial activity against *E. coli* isolates alone or in combination with antibiotics. Vitamin C MIC against *E. coli* isolates ranged from 0.625 mg/ml to 2.5 mg/ml with the most effective concentration of vitamin C against most isolates at 1.25 mg/ml, similar results were found by Mahmood and Abdullah in Egypt, who reported that MIC of vitamin C was 5 mg/ml against all coliform species [18]. Also, our results were confirmed by previous findings that reported that MIC of vitamin C against gram-negative bacteria ranged between 2- 16 mg/mL [14, 20, 21].

There is no correlation between the antibacterial effect of vitamin C and antibiotic resistance. There was no difference between the MIC of vitamin C and different categories of antibiotic susceptibility. Our results indicated that vitamin C had an inhibitory effect on most isolates regardless of their resistance to antibiotics, these results were also confirmed by El-Gebaly et al. in Egypt [22].

In the current study, vitamin C combined with antibiotics was used against all study isolates. The FIC of the combined drug was determined. We noticed that vitamin C had a synergistic effect with most of the studied antibiotics (Levofloxacin, Meropenem Ceftazidime and Nitrofurantoin). The synergistic effect of vitamin C was previously confirmed by Mahmood and Abdullah in Egypt, who reported synergism between vitamin C and ciprofloxacin against ciprofloxacin-resistant *E. coli* strains [18] and by Khameneh et al. who reported the synergistic effect of vitamin C with antituberculous drugs against mycobacterium [23]. Kwiecińska-Piróg et al., Cursino et al., Srividya and Lakshminarasaiah also reported the synergism of vitamin C with different antibiotics [24–26].

In our study, it was found that vitamin C had an anti-biofilm effect on *E. coli* isolates where 0.312 mg/ml was the mean concentration for its anti-biofilm effect, similar results were reported by Shivaprasad et al.who detect that vitamin C-treated bacterial culture showed a 5.8 folds reduction in EPS production in comparison to control without vitamin C [4, 23].

By analyzing RNA from *E. coli* isolates before and after treatment with vitamin C, the current study determined the expression levels of beta-lactamases and biofilm encoding genes**.** The expression levels of most of the studied genes *(bla*_*SHV*_*, bla*_*TEM,,*_*bla*_*CTX-M,,*_* bla*_*NDM,,*_* bla*_*IMP,*_* las*R, and *lec*A) were significantly reduced after vitamin C treatment(*P* values ˂0.05). Shivaprasad et al. reported the same results where expression levels of all the tested genes needed for *E. coli* biofilm formation were significantly down-regulated after vitamin C treatment [4].

Vitamin C shows acceptable in vitro results as an antibacterial and anti-biofilm agent, this encouraged us to test its effect in vivo. This is the first study wherein we evaluated the in vitro and in vivo effect of vitamin C as an antibacterial and anti-biofilm agent against *E. coli* clinical isolates*.*

In our in vivo experiment, the efficacy of vitamin C treatment in rats after a UTI with *E. coli* showed that: administration of vitamin C alone or combined with antibiotics in the infected animal improved the inflammation and lowered the bacterial count. Moreover our data revealed that vitamin C has a synergistic effect with antibiotics as administration of both vitamin C and nitrofurantoin at lower doses produces a more powerful effect. Also our data confirmed the anti-inflammatory and antioxidant properties of vitamin c as the UTI + C, and UTI + N + C groups showed significant decreases in the inflammatory mediators (MDA, and NFkB) but increases in antioxidants (GSH, and TAC) compared to the UTI group. Abdelraheem et al. confirmed these results and reported that vitamin C had a significant improvement effect as an antibacterial and anti-inflammatory agent in treatment of pneumonia caused by *P. aeruginosa* in rats [27].

## Conclusion

Vitamin C shows good results as an antibacterial and anti-biofilm agent in vitro and in vivo alone or combined with antibiotics. Vitamin C should be routinely prescribed alone or with antibiotics as antibiotic modifier to treat UTI infections in clinical settings as this combination will shorten the antibiotic course and lower the antibiotic dose. We recommended further in vitro and in vivo studies to explore vitamin C's potential as a safe and effective antibacterial agent in the fight against rising antimicrobial resistance as there are only a few studies on vitamin C compound toxicity. As well as a large-scale investigation on a large number of *E. coli* clinical isolates and other *E. coli* genes are recommended to investigate the effect of vitamin C on biofilm formation and antibiotic resistance.

## Methods

### Bacterial strains

The study was carried out on 100 non-repeated clinical isolates of *E. coli* obtained from patients with urinary tract infection at Minia University Hospital. Identification of *E. coli* isolates was performed by colony morphology on MacConkey and XLD agar (Oxoid, England), Gram staining, and standard biochemical tests (Oxoid, England) that is: triple sugar iron, sugar fermentation, indole, methyl red, vogues proskauer, citrate and motility tests, according to Cheesbrough methods and interpretations [28].

#### Antimicrobial susceptibility testing of antibiotics and vitamin C

Antimicrobial susceptibility testing and MIC of antibiotics and vitamin C against all isolates were determined by the micro-dilution method. The selection of antibiotics and interpretation of results was done according to CLSI, 2020 guidelines [29]. The following antibiotics were chosen: Ampicillin-sulbactam, Cefazolin, Cefoxitin, Ceftazidime, Meropenem, Levofloxacin, and Nitrofurantoin.

Stock solutions of antibiotics were prepared by dissolving pharmaceutically purchased antibiotics (PHARCO B International, Egypt) in sterile distilled water (1 mg/ml). The stock solution of ascorbic acid was prepared by dissolving commercially purchased vitamin C (L-Ascorbic acid, Sigma) in sterile distilled water (10 mg/ml). The range of the twofold serial dilutions was made for each antibiotic below and above the sensitivity breakpoints according to CLSI, 2020 guidelines. The twofold serial dilutions of vitamin C were between 5 to 0.019 mg/ml.

Two-fold serial dilutions of antibiotics or vitamin C were prepared in a 100 μl volume of Mueller–Hinton broth (MHB) in the MTP wells from well 1 to well 9, so there were 9 folds serial dilutions of each antibiotic or vitamin C. 10 μl of bacterial suspension, diluted according to CLSI protocol (10^5^ CFU/ml) was then inoculated in the wells. Wells containing an identical amount of basal medium with (positive control) and without (negative control) bacterial suspension, but free of antibiotics, and wells separately containing the antibiotic or vitamin C (drug control) were included in each assay as growth controls. The plates were incubated in an aerobic incubator for 20 h at 37 °C. MIC was visually determined and interpreted according to CLSI, 2020 guidelines. The MIC was defined as the minimal concentration of an antimicrobial that visually inhibited the bacterial growth after 20 h of incubation and was reported by observing the visual turbidity of the wells [30].

### Time kill-kinetics assay

Bactericidal activity is defined as a higher than 3 log_10_ fold decrease in colony forming unit (99.9 killing of the starting inoculum). The time-kill assay can monitor the effect of various concentrations of antimicrobial agents over time concerning the different stages of bacterial growth. The time-kill kinetics of vitamin C was done according to CLSI guidelines. Briefly, a grown culture of Muller Hinton broth with pure bacterial colonies (1.0 × 10^6^ CFU/mL) was inoculated with ascorbic acid at MIC and double MIC. Serial dilutions were done at each time point (0, 4, 8, 12, 16, 20, and 24 h) and subcultures on Muller Hinton agar plates were incubated at 37 ºC. A positive control was performed for the tested strains without vitamin C. A graph of log CFU versus time was created [31].

### Assessment of the combined effect of vitamin C with antibiotics:

The effect of the combination of vitamin C and antibiotics on the resistant strains of the corresponding antibiotic was assessed by the micro-dilution method. Briefly, two-fold serial dilutions of antibiotics were prepared in 100 μl volume of MHB in MTP wells. Half the original MIC of vitamin C in 100 ul volume distilled water was added to all wells of the MTP, so the final concentration of vitamin C in all wells was 1̸4 the original MIC. The procedure was completed as mentioned above. MIC of combined agents was determined and compared with the MIC of antibiotics alone.

Fractional inhibitory concentration index (FICI) analysis: FICI was used to calculate synergism, indifference, or antagonism between two compounds according to the following equation:

$$\mathrm{FICI }= {\mathrm{FIC}}_{\mathrm{A}} + {\mathrm{FIC}}_{\mathrm{B}} = ({\mathrm{C}}_{\mathrm{A}}/{\mathrm{MIC}}_{\mathrm{A}}) + ({\mathrm{C}}_{\mathrm{B}}/{\mathrm{MIC}}_{\mathrm{B}})$$

"CA" refers to the MIC value of drug A in combination with drug B, where "MICA" refers to the MIC of drug A alone. "CB" refers to the MIC value of drug B in combination with drug A, where "MICB" refers to the MIC of drug B alone. The FIC index value ≤ 0.5 indicates synergy, > 0.5–4 indifference, and > 4 antagonism [32].

### Anti-biofilm effect of vitamin C

All *E. coli* isolates were tested for biofilm formation before and after treatment with the sub MIC of vitamin C by the Micro-titre plate method for biofilm detection [26]. The MTP wells were inoculated with bacterial suspension (with 0.5McFarland turbidity) with and without serial dilutions of vitamin C. The MTP was incubated at 37 °C for 24 h. After incubation, the contents of the wells were decanted into a discard container and washed with saline then emptied by flicking the plate gently. The MTP was air dried at 60 °C for 60 min. The wells were stained with 150 μl of 0.2% crystal violet solution for 15 min. The stain was discarded and wells were washed with water. The stained wells were solubilized with 95% ethanol. The absorbance of each well was measured at 590 nm by an ELISA reader (model CS, Biotec).

### Effect of vitamin C on gene expression

All beta-lactam resistant and biofilm-producing *E. coli* isolates were analyzed for the expression level of beta-lactamases encoding genes (*bla*_*SHV*_*, bla*_*TEM,*_* bla*_*CTX-M,*_* bla*_*VIM,*_* bla*_*NDM,*_* and bla*_*IMP*_) and biofilm-associated genes (*las*R, *lec*A and *pel*A) respectively, before and after treating the isolates with vitamin C by relative gene expression method. It is a valuable method of quantifying gene expression by comparing target transcripts and reference genes. Relative gene expression calculations for the tested genes with *rpsl* gene as a reference gene were performed twice before and after in vitro treatment with vitamin C for all tested isolates by RT-PCR according to the following steps.

#### RNA extraction

All tested *E. coli* isolates were inoculated in nutrient broth. The turbidity of bacterial broth was adjusted to the turbidity of 0.5 McFarland standards. Two tubes for each isolate were prepared and sub MIC of vitamin C was added to one of them. All tubes were incubated for 24 h at 37 °C. After incubation, the tubes were centrifuged (13,000 rpm, 10 s) and the deposit has been used for RNA extraction according to the Direct-zol RNA extraction kit protocol (Zymo research CORP, Australia). The purity and concentration of the extracted RNA were evaluated by measuring the absorbance of the RNA using a spectrophotometer (Genova, USA). The quality of the extracted RNA was assessed via gel electrophoresis at 100 V.

#### Real-Time Reverse Transcriptase-Polymerase Chain Reaction (RT-PCR) step

Real-Time PCR was performed on the extracted RNA using one-step Sybr green kits according to manufacture instructions (Sensi FAST SYBR Lo-ROX one-step Kit (code noBIO-74005). Each PCR reaction was prepared with a final volume of 20 μl (master mix: 10 μl, Forward primer: 0.8 μl, Reverse primer: 0.8 μl, Reverse transcriptase: 0.2 μl, RNase inhibitor, 0.4 μl, Water up to 16 μl and template: 4 μl). Each sample was tested out three times in three independent experiments and the average was taken Negative control samples containing deionized water were used with each run. The PCR conditions were adjusted according to the kit protocol as follows: reverse transcription for 10 min at 45 °C, Polymerase activation for 2 min at 95 °C, denaturation (40 cycles) for 5 s at 95 °C, and annealing/extension for 20 s at 60 °C. The PCR products were analyzed by gel electrophoresis, to exclude any unspecific products. The primer sequences used for amplification of the studied genes were taken from previous published studies [33, 34].

##### Interpretation of the results

The cycle threshold (CT) (the number of cycles required for the fluorescent signal to cross the threshold line in real-time PCR) was recorded for the target genes and reference gene to calculate the fold change (relative quantity “RQ”). The relative expression of target genes was calculated using the comparative cycle threshold (ΔΔCt) where, ΔCt = Ct (gene of interest)—Ct (reference gene) and ΔΔCt = ΔCT of the test sample –Δ CT of the control sample. RQ = 2 − ΔΔCt [35].

### In vivo assessment of the antibacterial effect of vitamin C

#### Rats

Male Wistar rats weighing 250–280 g were obtained from National Research Center (Giza, Egypt). The 30 rats were divided into five groups (6 rats each), group one (Nitrofurantoin, N), group two (vitamin C; C), group three (vitamin C + Nitrofurantoin, C + N), group four, (UTI) and group five is the normal control group (Con).

All rats in all groups except the normal control group were trans-urethrally inoculated with *E. coli* clinical isolate The UTI rat model was based on that of Hannan with some modifications. The rats were anesthetized with ketamine. Anesthetized rats were inoculated trans-urethral with the bacterial suspension by the intravenous gauge (0.8 mm) as a catheter. The tips were perfectly smooth so that they did not cause tissue injury during inoculation. The catheter was carefully pushed in horizontally until it reached the top of the bladder, and 0.1 mL of bacterial suspension was injected into the bladder using a tuberculin syringe with 10 μl gradations over 5 s to avoid vesicoureteral reflux. The catheter was removed immediately after inoculation. Forty-eight hours after inoculation, treatment has been started. Treatment options are given by oral route for groups: 1, 2, and 3 for 5 days. Group 1 was treated with nitrofurantoin antibiotic (100 mg/kg/day), Group 2 was treated with vitamin C (200 mg/kg/day) and Group 3 was treated with nitrofurantoin (50 mg/kg/day) and vitamin C (100 mg/kg/day) for 5 days. Group 4 didn't receive any treatment [36].

#### Sample collections

Urine samples were collected in a sterile Eppendorf tube before bacterial inoculation (day zero) and then daily during the study period. At first we determined the PH of urine samples using commercial urine strips, and then bacterial count was done. Two rats from each group were culled 2 days after treatment (four days after inoculation) then the remaining rats were culled 5 days after treatment. Blood samples were collected from the abdominal aorta for serum separation. The kidneys and bladders were aseptically removed and used for bacteriological, biochemical, and histological examination.

#### Bacterial count

The kidney and bladder were aseptically homogenized in sterile saline using tissue homogenizers. 100 μl volumes of tenfold serial dilutions of the tissue homogenate and urine were cultured on solid media. The bacterial count was calculated per 1 ml of urine or per gram of tissue.

#### Evaluation of oxidative stress parameters (MDA, GSH, TAC) and NFkB

The most reliable index of lipid peroxidation is Malondialdehyde (MDA). It was measured according to Buege's method. In addition, measurement of reduced glutathione (GSH) was determined calorimetrically on basis that Ellman’s reagent could reduce the thiol group of GSH and yellow color is formed and detected by spectrophotometer at 412 nm.

The serum levels of TAC and tissue level of NFk B were quantified according to the manufacturer's instructions [37, 38].

### Statistical analysis

Statistical analysis for the in vitro test was done by SPSS package version 23.0 (SPSS Inc., Chicago, IL, USA). Chi-squared tests were performed for categorical data; while Wilcoxon Signed Rank tests were performed for the comparison of continuous data. Statistical analyses for the in vivo results were performed using the Graph Pad Prism Version 9.0 (Graph Pad Software Inc. San Diego, CA, USA). Data are presented as means ± SEM unless otherwise specified. Differences in quantitative measures were assessed by Student’s *t*-test or one-way ANOVA followed by Turkey's multiple comparison tests, when appropriate. Differences were considered significant when the *P* value < 0.05.

## Supplementary Information

**Additional file 1: Supplementary figure S1.** The effect of different vitamin C on biofilm formation. NC is the negative control. The lowest concentration that inhibits biofilm formation is 0.312mg/ml.

## Data Availability

All data generated or analyzed during this study are included in this article.
