# Supplementary material for: Antibacterial effect of vitamin C against uropathogenic E. coli in vitro and in vivo
Source: BMC Microbiol. 2023 Apr 20;23:112. doi: 10.1186/s12866-023-02856-3 (PMC10116447; doi:10.1186/s12866-023-02856-3)
Supplement: Supplementary file 1 — Additional file 1: Supplementary figure S1. The effect of different vitamin C on biofilm formation. NC is the negative control. The lowest concentration that inhibits biofilm formation is 0.312mg/ml. [file 12866_2023_2856_MOESM1_ESM.docx]

Supplementary figure


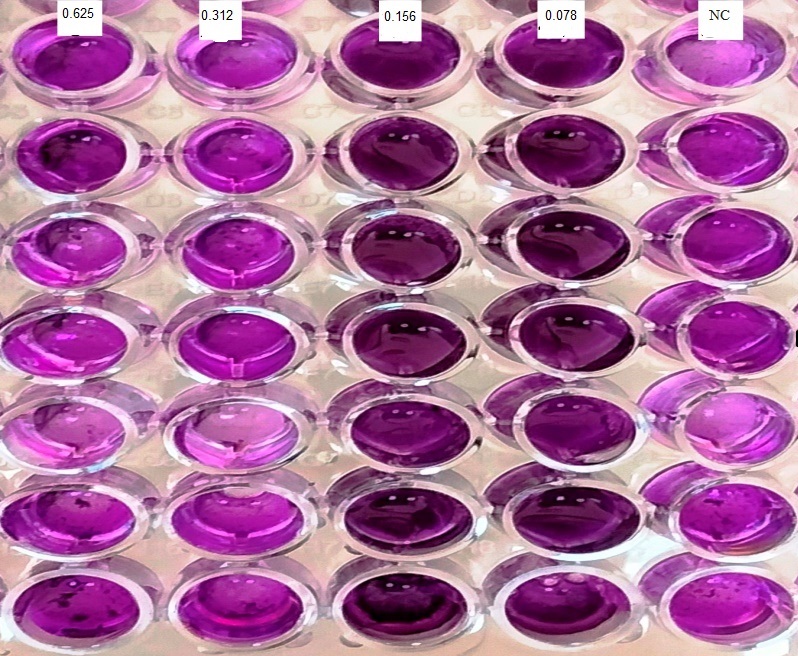


Supplementary figure S1: The effect of different vitamin C on biofilm formation. NC is the negative control. The lowest concentration that inhibits biofilm formation is 0.312mg/ml.
